# Remote Sensor for Spatial Measurements by Using Optical Scanning

**DOI:** 10.3390/s90705477

**Published:** 2009-07-10

**Authors:** Oleg Sergiyenko, Wilmar Hernandez, Vira Tyrsa, Luis Felipe Devia Cruz, Oleg Starostenko, Mario Peña-Cabrera

**Affiliations:** 1 Engineering Institute, Autonomous University of Baja California, Calle de la Normal S/N, col. Insurgentes, Mexicali, Baja California, Mexico; 2 Department of Circuits and Systems in the EUIT de Telecomunicación at the Universidad Politécnica de Madrid (UPM), Campus Sur UPM, Ctra. Valencia km 7, Madrid 28031, Spain; E-Mail: whernan@ics.upm.es; 3 Universidad Politécnica de Baja California, Mexico; E-Mail: vera-tyrsa@yandex.ru; 4 Universidad de Las Americas, Puebla, Mexico; E-Mail: oleg.starostenko@udlap.mx; 5 Instituto de Investigaciones en Matemáticas Aplicadas y en Sistemas (IIMAS), Ciudad Universitaria, Univesidad Nacional Autónoma de México, D.F. CP 04510, Mexico; E-Mail: mario@leibniz.iimas.unam.mx

**Keywords:** passive scanning, health monitoring, robot navigation, laser

## Abstract

In this paper, we propose a low-cost contact-free measurement system for both 3-D data acquisition and fast surface parameter registration by digitized points. Despite the fact that during the last decade several approaches for both contact-free measurement techniques aimed at carrying out object surface recognition and 3-D object recognition have been proposed, they often still require complex and expensive equipment. Therefore, alternative low cost solutions are in great demand. Here, two low-cost solutions to the above-mentioned problem are presented. These are two examples of practical applications of the novel passive optical scanning system presented in this paper.

## 1. Introduction

Optical scanning is a very common method to obtain fast, safe and cheap information. In this paper a novel passive optical scanning method is presented.

### 1.1. Large civil structures health monitoring

As we know, today’s civil infrastructures consist of various units and systems such as buildings, bridges, hydroelectric plant dams, offshore platforms, transportation and communication systems, and so on; and a significant amount of expenditures and long working periods have been needed for our society to build up its infrastructure. The satisfactory performance of our infrastructure and systems is vital for our economic activity [1,2].

Buildings and, in general, infrastructures have an enormous impact on the quality of the environment. However, if they are degraded by improper maintenance, they can become very expensive. To prevent this, it is necessary to develop maintenance technology for inspection, monitoring, repair, rebuilding and carrying out financial planning recommendations.

Structural health monitoring (SHM) is one of the most important components in the maintenance technology for civil infrastructures and, until now, some methods of structural monitoring have been used successfully, while recently proposed methods offer the possibility of extending applications and improving efficiency.

Damage in a structure generally causes a local increase in flexibility, which depends on the extent of the damage. Current methods of damage detection include visual inspections, classical nondestructive techniques (NDT) and vibration/modal-based methods [1–3]. Most of the NDT techniques are based on regular ground inspections which are time consuming and expensive. Typical NDT methods for damage evaluation include: sonic, ultrasonic, acoustic-ultrasonic, acoustic emissions, pulse-echo, x-ray imaging, ground penetrating radar (GPR), and dye penetrant inspection to detect cracks [4].

Global Positioning System (GPS) technology, along with the use of Pseudo-Satellites, has been widely used for monitoring the deformation of civil structures. However this technology has some drawbacks, namely the high cost of receivers and pseudo satellites and it cannot be used when the signals are completely blocked by either natural or man-made obstacles [5].

Recent advances in the development of new techniques involve the use of embedded smart sensor and actuator technology in order to reduce the need for visual inspection, to assess structural integrity and mitigate potential risk in large civil structures such as highways, bridges and buildings.

These sensors and actuators are typically made of a variety of materials including piezoelectric and magnetostrictive materials, shape memory alloys, electrorheological and magnetorheological fluids, fiber optic sensors, and so on. These materials can typically be embedded into the host matrix material of the structure in order to either excite or measure its state [6,7].

Most of today’s bridges and roads do not have built-in “intelligence”. Therefore, they cannot take advantage of the benefits of the advanced technologies available for SHM, and lots of them are in bad conditions due to inadequate maintenance, excess loading (relative to their original design and expected usage), and adverse environmental conditions (salt, acid rain, etc.).

In this paper, the design and potential of passive optical scanning (POS) aperture in deformation monitoring of civil structures is discussed. In short, POS is based on optical exploration of luminous reference points previously fixed to the civil structure, and in order to determine the damage level of the structure, the coordinates are periodically compared with the values contained in a historical information data base.

### 1.2. Vision system for mobile robot navigation

Position determination for a mobile robot along with the ability to measure surfaces and objects in 3-D is an important part of autonomous navigation and obstacle detection, quarry mapping, landfill surveying, and hazardous environment surveying.

In spite of the fact that a significant number of dead reckoning systems use landmark recognition, either by extracting relevant natural features (corners, objects, etc.) using a camera or by identifying beacons placed in some specific places using both a camera and an optical scanner, in many cases dead reckoning is insufficient because it leads to large inaccuracies over time.

Beacon- and landmark-based estimators require the emplacement of beacons and the presence of either natural or man-made structures in the environment [8], and due to the fact that image processing consumes an amount of time that cannot be discarded and requires positioning accuracy as well, it cannot satisfy the autonomous navigation in many cases. Major drawbacks of beacon-based navigation are that the beacons must be placed within a range and that they must be appropriately configured in the robot work space.

The GPS based navigation for autonomous land vehicles has the capabilities to determine the locations of vehicles based on satellites. Although commercial Differential GPS (DGPS) service increases the accuracy to several meters, it is still not sufficient for autonomous driving. It is well known that with the different types of GPS systems that exist we can obtain positions with errors from 0.02 m to l m. Nevertheless this accuracy cannot be guaranteed all the time in most working environments, where partial satellite occlusion and multipath effects can prevent satisfactory GPS receiver operation [9–13]. Environmental sensors, such as 3-D scanning laser rangefinder and ultrasonic environmental sensors, are used as well.

Given the current state-of-the art of this topic [13–15], we believe that a 3-D laser scanner would be the best choice for building 3-D maps. However, 3-D scanners are expensive solutions for most mobile robot applications, and lots of them are not fast enough for real-time map building on fast-moving vehicles, due to the relatively slow vertical scan.

A cost-effective alternative for 3-D mapping is to mount a 2-D laser scanner on the front-top of a mobile robot. During motion, the fanning beam of the scanner sweeps over the terrain in front of the robot, effectively creating a 3-D map.

Triangulation-based laser range finders and light-striping techniques are well-known techniques from more than twenty years ago, and contrary to other active techniques such as structured light, coded light, time of flight, etc., laser range scanners are commonly used for contact-less measuring of surfaces and 3-D scenes in a wide range of applications.

Most commercial laser scan systems use both a camera and a laser beam or laser plane, the surface recovery is based on triangulation (i.e., the intersection of the illuminating laser beam and the rays projected back to the camera [12]) and expensive high-precision actuators are often used for rotating/translating the laser plane or for rotating/translating the object.

In this paper, a scanning vision system that overcomes the above limitations and provides a system that will meet the existing demand for more advanced mobile vehicle navigation systems has been designed.

The system has been developed for visual inspection tasks in both indoor and outdoor environments; and the proposed POS system along with a Laser Positioning System (LPS) allow us to obtain 3-D information of possible obstacles in front of a mobile robot, to make intelligent decisions and to carry out tasks of automatic navigation. This paper also focuses on some of the design and application issues of the POS aperture.

Finally, it is important to highlight that a novel passive scanning aperture (PSA) device is introduced in this paper in order to increase spatial resolution, as the central part of a proposed system with the technical tasks of SHM and robot vision.

## 2. Problem Statement

**Figure 1.**
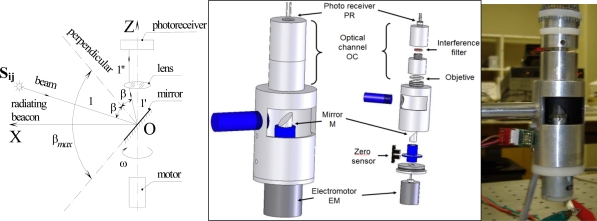
POS system.

The foundations to solve the tasks of navigation and civil structures monitoring, are the creation of the POS (see Figure 1), which has been developed to detect incidence light angle from monitored structures [14–19].

In general, this device is based on the principle of the automated theodolite and, due to its optical design, it allows capture of the same-rate optical data from the active targets located at different heights in the pre-defined field-of-view (FOV). Also, owing to the fixed distance (focal distance F) between the mirror M and the objective lens, the POS is able to register uniform signal amplitude in the photoreceiver circuit. This allows it to obtain the same coordinate accuracy for points located at different heights within desired FOV.

The POS must be placed in a fixed location in the robot or the SHM system, depending on the application, and it consists of a fixed assembly to support and align the electro-optical elements of the system. The optical elements are the following: double-convex lens, an interference filter and a 45° cylindrical mirror microrod.

**Figure 2.**
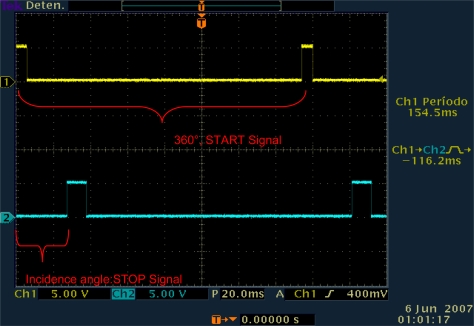
Electric signals generated by POS system on spot and synchronous point detection.

The mirror is mounted on the shaft of a direct current (DC) electric motor (EM) (see Figure 1) that turns the mirror around on its own vertical axis. It has two sensors, an opto-switch to indicate the starting point of the turn of the mirror (START signal) and a photodiode to detect light emission points (STOP signal) (see Figures 1 and 2) [14–18].

The main task of the POS is to conduct a search in the horizontal plane of the light emission points S_ij_ (see Figure 1), which represent the (*i*,*j*)-position of the active target during the scanning (*i*: length, *j*: height), that are within its FOV. These emission points either can be fixed previously on a study surface or can be reflection points of light on a surface.

The principle of operation of the POS is as follows: the mirror rotates at a constant speed and whenever it passes over an initial reference point, the starting point of the rotation of the mirror (START signal; see Figure 2), a START signal is generated starting the pulse counting of a constant high-frequency reference signal *f_o_*, until the instant of time at which the scanning plane coincides with the emitting point S_ij_.

Figure 2 corresponds to a typical screenshot of the Tektronix TDS-3012B two-channel oscilloscope connected to the zero-sensor circuit and the photo-receiver circuit of the prototype under real-time operating conditions. In this case, by scanning plane we mean the plane formed by two intersected straight lines, the motor rotation axis and the gravitational force line projection on the plane of mirror. When the point S_ij_ belongs to this imaginary scanning plane, the STOP pulse is formed. Only at this short instant of time, when the check point S_ij_ coincides with the scanning plane, there exists the physical possibility of carrying out the transmission of a certain part of the light energy emitted by S_ij_. When this happens, the ray caught by the mirror is reflected with the same angle as the one it arrived at the double-convex lens, and it passes through the interference filter until the moment at which it arrives at the photo-detector, generating a STOP signal.

In the case that there is no signal, if during a complete rotation of the mirror an emitted ray of light is not detected, then the counter of pulses of *f_o_* will be reset.

In this process we can find the horizontal coincidence angle B*_i_* between the START signal and the STOP signal,

(1) $${Β}_{i}=2\pi \cdot N_{\mathrm{Bi}}/N_{2\pi 1}$$

where *N_Bi_* is the number of pulses of *f_o_* between the START and the STOP signals, and *N_2π1_* is the number of pulses of *f_o_* in a complete rotation of the mirror. These codes depend on the rotation speed of the motor *ω* and the frequency *f_o_* [19].

## 3. Practical Applications of the Proposed Method

### 3.1. Monitoring of civil structures

One of the applications of the POS is in monitoring of civil structures for determining the health of such structures [20]. These structures might be sufficiently different from one another, and the proposed system configuration can be slightly modified in order to obtain the desired data from the surface of the structure in an optimal way.

#### 3.1.1. Bridge monitoring

**Figure 3.**
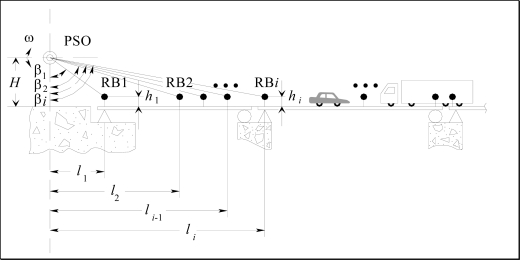
Horizontal POS placement for bridge monitoring.

As an example we consider the bridge shown in Figure 3. Here it is necessary to install Radiating Beacons (RB) on the surface of the structure at the same height *h_i_* The distance *l_i_* between each RB is measured during the installation, and in order to measure the horizontal and vertical displacements of each RB, two POS are installed: one POS to measure horizontal angles (HPOS) with vertical axis rotation and the other POS to measure elevation angles (VPOS) with horizontal axis rotation.

Then, the following expression is valid for each RB_i_ from *i* = 1 to n when there is no deformation:

(2) $$\frac{H-h_{i}}{l_{i}}=\text{tan}(\frac{\pi }{2}-\beta_{i})$$

When there is a deformation with magnitude Δ*h_i_* at the point RB_i_, the measured angle by the POS *β_i_* changes in magnitude Δ*β_i_* and the expression (2) is given by:

(3) $$\frac{H-h_{i}\pm \Delta h_{i}}{l_{i}}=\text{tan}\left(\frac{\pi }{2}-\beta_{i}\pm \Delta \beta_{i}\right)$$

Therefore:

(4) $$\Delta h_{i}=(H-h)-l_{i}\;\text{tan}(\frac{\pi }{2}-\beta_{i}\pm \Delta \beta_{i})$$

In this case, the system does not have to be affected by the vibrations of the structure. The rotation velocity of the POS is fixed and based on the natural vibration frequency of the monitoring structure and on the distance “POS – structure” in order to be able to observe abnormal distortions.

#### 3.1.2. Arch dam monitoring

**Figure 4.**
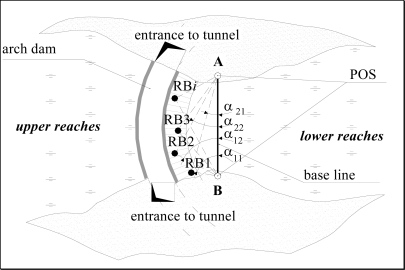
Placement of two vertical POSs for arch dam monitoring.

Figure 4 shows the placement of two vertical POSs for monitoring the arch dam of a hydroelectric power station. The POSs measures in pairs the horizontal angles *α*_1*i*_ and *α*_2*i*_ between the base line AB and RB_i_. The length of the base line *D* and the Cartesian coordinates of the POS are previously determined by known geodetic methods.

The angles ∠RB_i_ are calculated with the following equation:

(5) $$∠{\text{RB}}_{i}=180{}^{\circ}-(\alpha_{1i}+\alpha_{2i})$$

According to the sine theorem, we find the triangles sides with vertex A, B and RB_i_, and the *x_i_*, *y_i_* Cartesian coordinates of the points RB_i_. If there are changes in the RB_i_ coordinates, then we will register changes in the angles *α*_1*i*_ and *α*_2*i*_, which will indicate dam deformation. In a similar manner, the POS system is installed for tunnel monitoring. In extended tunnels, several functionally connected POS can be placed along its axis.

### 3.2. Scanning vision system for robot navigation

**Figure 5.**
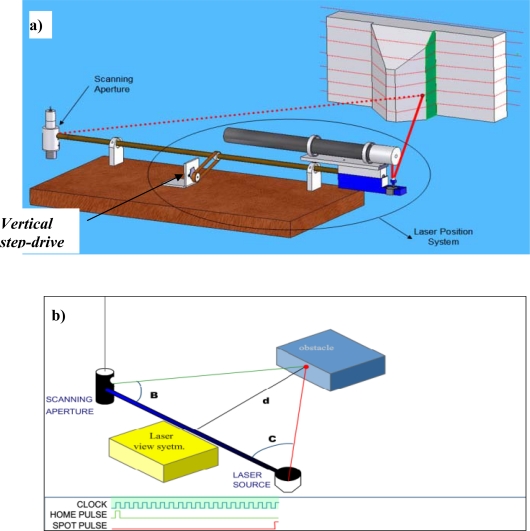
POS and LPS placement for automatic navigation task. a) General view of the system with interaction of the parts in functioning. b) Triangulation angles distance and signals from sensors and reference *f* (“clock” pulses).

The Scanning Vision system is another application of a POS [21–23]. In this case, it is only necessary to install one POS with a scanning LPS. Figure 5 shows a simple diagram of a triangulation scanner. The laser beam -reflected from a mirror in the LPS- is projected on the object. The diffusely reflected light is collected by the POS if laser spots are projected.

The laser positioning circuitry (see Figure 5) controls both the emission angle C_ij,_ and the elevation angle Σβ_i_. The elevation angle is not shown in Figure 5; however, it is calculated exactly in the same way that the angle C_ij_, by direct count of steps of vertical step-drive (see Figure 5 a)). Both angles can be calculated in the same manner. The angle B_ij_ is calculated by the POS measurements; and the triangulation distance, the distance ***a*** between the centers of POS mirror and the LPS mirror [see Figure 5 b)], is also known. In other words, the distance ***a*** is the same as the base-bar length.

As shown in Figure 5, since all geometric parameters are known, the *x*, *y*, *z* coordinates of the point S_ij_ on the object can be computed in trigonometric form. If a single laser dot is projected, the system measures the coordinates of just one point of the object [19]. When a laser discrete stripe is projected, all points along the stripe are digitized. The basic triangulation scheme can be enhanced to improve the optical quality and field depth by simple variation of “motor step size/steps quantity”.

If we place the fast-operation POS on autonomous mobile objects, such as robots, vehicles, transportation carriage, military devices, and so on, we can use it as an artificial vision system for automatic navigation task solutions.

## 4. Experimental Results of the Prototype

**Figure 6.**
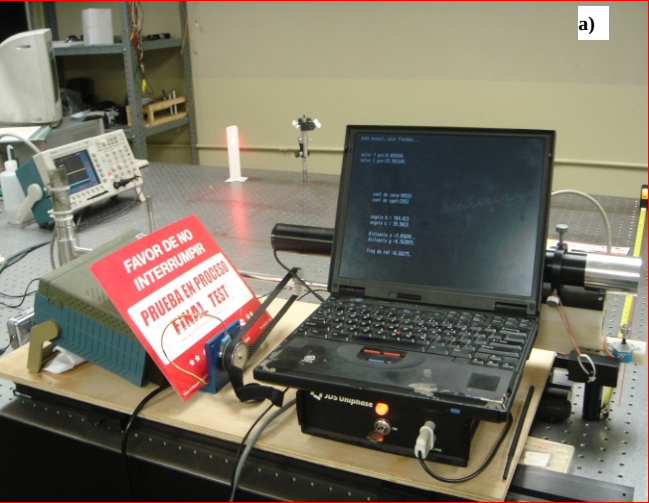

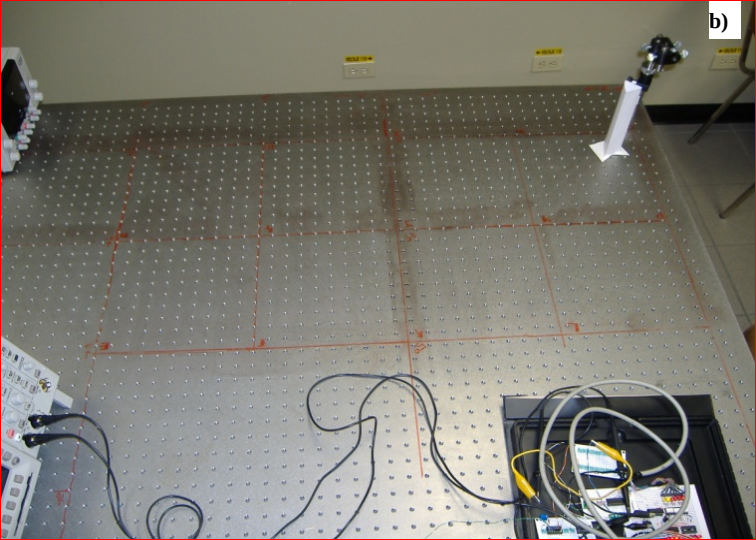
Experiments using laser spot: a) on obstacle variable position. b) in order to determine coordinates (*x,y*).

The system shown in Figure 5 was designed and constructed for laboratory tests [22–25]. The experiment to obtain the coordinates (*x,y*) of an illuminated point into scene was carried out by using a JDS Uniphase Model 1135P laser source (20 mW; He-Ne; see Figure 6).

Parameters of the prototype:

- sampling rate: 231 points per image in a fixed FOV of 1 × 2 m with a reference pulse train of 114.675 kHz.
- rotation speed: For the JAMECO DC motor MD5-2554AS-AA the speed of rotation is 13,860 rpm (231 rps) at 17,000 rpm, output torque is equal to 72.92 g·cm. But in practice, it was reduced to 7–13 rps due to time response of selected photosensor.
- dynamic range: 7–13 Hz with 114.675 KHz reference frequency at maximum speed.
- spectral range: λ = 625–740 nm for sensor, with peak value nearby laser bandwidth, and 633 nm for laser.
- spatial resolution: For a distance of 2.5 m on the optical table, it was 0.001–0.01 of the x,y-value in the best points, in the center of the FOV, and 0.05–0.08 of the x,y-value in the worst points, at the edge of the FOV. Detailed experimental data are presented in [22] and [23].

**Figure 7.**
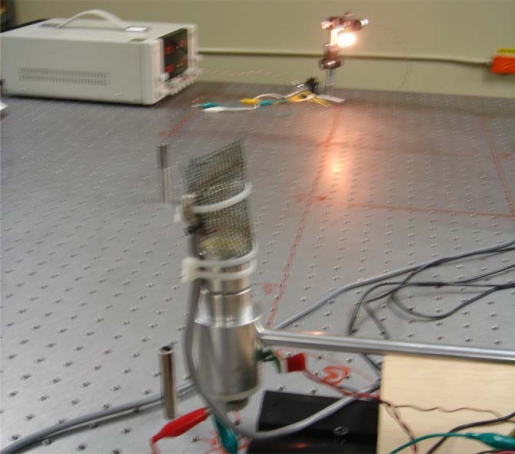
90° angle measurement using a light bulb as the energy source.

The experiments to test the accuracy of the measurement of the incidence angle were carried out by using an active target in a form of light bulb, in this case a 12 VDC 50 W incandescent lamp, see Figure 7. However, for an increase in the striking distance it is still possible to use a LED or a super-bright LED.

Experimental data based on 200 samples of 45° and 90° measurement angles are shown in Table 1.

**Figure 8.**
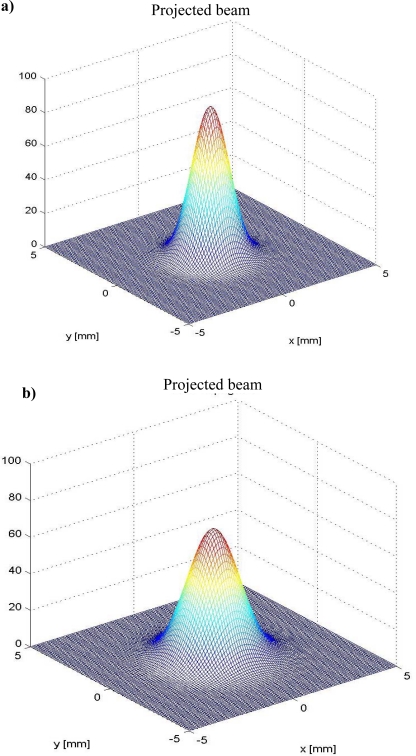
Signal energy attenuation and distribution in projected spot: a) striking distance 2.5 m. b) striking distance 5.0 m.

From Figure 8 it can be seen that the optical signal amplitude is sensitive to both rotation velocity and striking distance, and that this relation is strongly non-linear.

For each one of the 2.5 m intervals within the expected striking distance 0–100 m, the computational model for a spatial intensity attenuation of the radiated light from 0% to 95% was simulated by using the Matlab software Poon. Since the experiments were limited in practice by the geometrical size of the optical table (see Figures 6 and 7), Figure 8 shows radial power distribution of laser beam for the revised cases of striking distance 2.5 m and 5.0 m, respectively.

As it can be seen from Figure 8, the registered light emission converted to an electrical signal by the photoreceiver (Silicon Detector 15.0MM2, Edmund Optics) can vary significantly, even at small distances. Due to this reason it was also expedient to carry out additional experimental research on these limitations for declared practical applications. For the task of navigation, from 5 m to 10 m, the performance of the proposed configuration was satisfactory.

**Figure 9.**
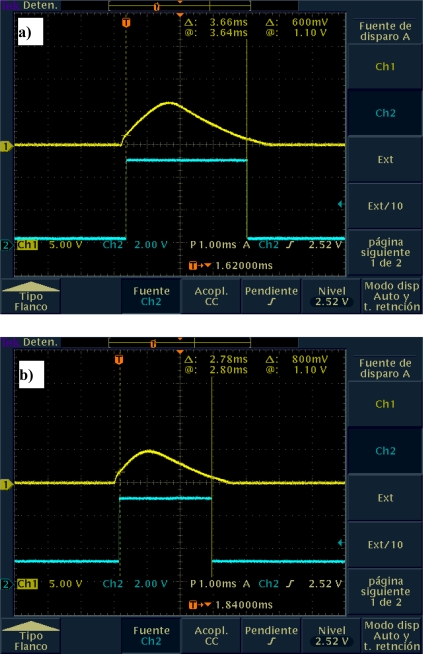

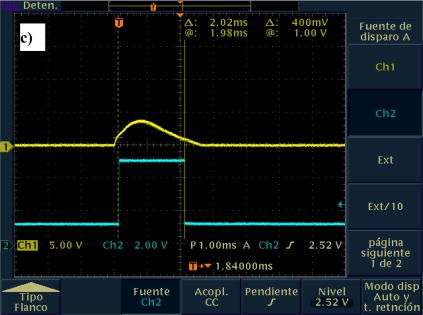
POS output signal amplitude variation according to rpm on mirror motor.

The output signal of the POS was analyzed in order to determine the dynamic limits of this system. Figure 9 shows changes in the amplitude of the output signal of the POS system with respect to changes in the speed of the DC motor.

As the speed of the DC motor increases, the signal amplitude decreases due to the capacitance of the photodiode model [14–18]. According to Figure 9, for a scanning velocity variation from 2–3 rps up to 25 rps, there are variations in both the pulse amplitude (from 400 mV up to 1.10 V) and the pulse width (from 2.02 ms up to 3.66 ms). Such variations can be easily corrected by using signal conditioning techniques. However, as can be seen from the above mentioned reasons, for SHM tasks these speed parameters are already acceptable for normal operation.

## 5. Conclusions

In this paper, in order to carry out 3-D object recognition, a contact-free measurement system for object surface recognition has been presented. Such a system has the following advantages over other 3-D triangulation scanners:

- It requires neither expensive non-telescope tubes for scanning ray positioning nor mega-pixel sensors matrix.
- It can be adjusted to be used in a wide range of practical applications due to the ability to change the step-size of the emitter subsystem for each specific task.

The results of the application of the remote precise optical-scanning system presented in this paper have been satisfactory.

## Figures and Tables

**Table 1.** Statistical data for 45° and 90° measurement angles.

	90°	45°
Average	90.278	45.316
Error t	0.142	0.035
Median	90.201	45.284
standard deviation	1.419	0.354
Variance	2.014	0.125
Asymmetry coefficient	0.302	0.080
Range	7.168	1.984
Min. Value	86.495	44.296
Max. Value	93.663	46.280
Samples	100.000	100.000
